# Global Mental Health: how do we make it ‘global’?

**DOI:** 10.1017/gmh.2014.4

**Published:** 2014-09-11

**Authors:** Florence K. Baingana

These are exciting times for global mental health. The momentum to get mental health better positioned in the global health agenda is picking up. Key indicators of this include, but are not limited to:

• World Economic Forum Report, which made a stronger case for the economic burden of non-communicable disorders (NCDs), including mental health. It is estimated that over the next 20 years, NCDs will costs more than 30 trillion dollars, with mental disorders contributing an additional 16 trillion dollars (Bloom *et al.* 2011).
• In 2011, the NIMH made the first RFA for the Collaborative Hubs for International Research in low- and middle-income countries (LMIC).
• Since September 2012, Grand Challenges Canada (GCC) have already committed 31.5 million to *Global Mental Health* in 49 projects in LMIC. GCC is one of the first granting agencies to have as a requirement LMIC Principal Investigators.
• World Health Assembly Resolution 65.4 of 2012, titled ‘The global burden of mental disorders and the need for a comprehensive, coordinated response from health and social sectors at the country level (WHO, 2012)’.
• The following year (2013), the WHO Twelfth Programme of Work included mental health as one of the priorities for the 2013–2016 triennium (WHO, 2013).
• The same year (2013), the theme for the Commonwealth Health Ministers Meeting was ‘Mental Health: Towards economic and social inclusion’ (Commonwealth Secretariat, 2013).

These are indeed exciting times, however, what changes can we hope to actually see, and what knowledge will in fact be translated into action especially to make an impact in low-resourced settings? How do we ensure that the gains made with an increased policy focus on mental health at the global level, as well as increasing funding to mental health research in areas with serious treatment gap challenges, translate into improved mental health services and population benefit? How does the global research agenda progress to advance these objectives and this impact? *Global Mental Health* will actively address discussions of future needed directions for the global mental health evidence base as well as about who is shaping and using the evidence base, in general. But my Associate Editor role at *Global Mental Health* reflects a purposeful effort to ensure that the perspectives from low and middle income countries (LMIC) are considered.

The Editors at *Global Mental Health* recognise that uptake of research evidence is determined by who makes contributions to this ‘evidence’. We will be mindful of questions such as how relevant is this research evidence to LMIC countries, who carried out the research, how were the research questions determined and what is the contribution of LMIC researchers, implementers and policy makers?

While the journal will have a focus on interventions, aetiology, policy and systems as well as teaching and learning, from a global perspective, *Global Mental Health* will make explicit this effort to focus on the LMIC perspective in these areas. The aim is to publish a journal that is truly global in nature. Every effort is going to be made to cultivate and encourage contributions by LMIC researchers, including reporting on issues that are pertinent to this readership.

There is evidence to suggest that there is a dearth of mental health services research by LMIC researchers. This is more so for some areas than for others. Although there is some research going on in the clinical services area, there is very little mental health systems and services research. A quick search of the NIMH website shows 120 clinical research and trials items and only 30 for mental health systems research. Among these, those from LMIC are even fewer. Yet, it is necessary to have health systems strengthened to deliver the clinical care. LMIC researchers are also not publishing. There is also substantial segmentation by target population that skews the issues relevant to LMICs, i.e. research and publishing around conflict-affected populations, or complex emergencies saw a huge upsurge in the 1990s and the early 2000s but have since declined.

The evidence for investing in mental health research is now strong, from basic sciences to evidence on how mental health services can be translated into policy. However, gaps in evidence still exist in how policy is translated into programmes (uptake), in LMIC. There seem to be two different gap areas in the area of translation: (i) it is not clear how much of research is translated into policy and how much of policy into programmes; and (ii) it is also not clear how much of this translation work is documented and published. In many LMICs, owing to limitations on resources, the focus is on implementation; often, the ‘how to’ of how evidence got into policy and the ‘how to’ of how policy translated into programmes, are not frequently published on, more so for mental health policy.

My role will be to encourage and coach contributions of researchers from LMIC, as well as support editorial and features content that addresses issues and challenges in building more LMIC-based research capacity and impact. Possible ways to foster contributions include disseminating as widely as possible the calls for the various issues that are to be published. This will be through the growing networks and mailing lists that I as well as our readers participate in. Another approach is to seek the views of LMIC researchers as to what they would like to publish on. This could be another way to get more LMIC researchers making contributions. Invited contributions from LMIC researchers, implementers and policy makers will stimulate more contributions from LMICs, as well as garnering their views as to what some of the challenges to *Global Mental Health* could be and how to overcome them. In some instances, such as right now as we debate the Post 2015 agenda, it is not clear whether the views expressed in international fora represent the views of LMIC. *Global Mental Health* intends to host topical discussions on such issues, and the views of LMIC stakeholders will be actively sought.

We will also seek the views of LMIC researchers, implementers and policy makers as to how to increase the contributions and the readership of *Global Mental Health*. A dialogue cannot be a dialogue if contributions are one way, but also if the readership and feedback is from HIC only. Since *Global Mental Health* will be Open access, and will waive publishing fees for contributions accepted through 2015. We hope this will encourage readership of LMIC stakeholders.

I am therefore very excited to be a part of this groundbreaking initiative, and to have this role within it. I am sure there may be other approaches that potential authors and readers of *Global Mental Health* may suggest to increase the participation of LMIC, and we encourage this feedback.
